# Effects of Topical Tebufenozide Application to *Choristoneura fumiferana* Pupae (Lepidoptera: Tortricidae)

**DOI:** 10.3390/insects11030184

**Published:** 2020-03-14

**Authors:** Lucas E. Roscoe, Glen Forbes, Rosanna Lamb, Peter J. Silk

**Affiliations:** Canadian Forest Service – Atlantic Forestry Centre, Natural Resources Canada, 1350 Regent Street, Fredericton, NB E3B 5P7, Canada; glen.forbes@canada.ca (G.F.); rosanna.lamb@canada.ca (R.L.); peter.silk@canada.ca (P.J.S.)

**Keywords:** spruce budworm, adult fitness, insect growth regulator, laboratory tests

## Abstract

*Choristoneura fumiferana* (Clemens) (Lepidoptera: Tortricidae) is a defoliating pest in Canada and the northeastern United States. Given its important ecological and economic effects in affected regions, several direct management techniques have been developed, including the application of the insect growth regulator tebufenozide (Mimic™, RH-5992) to feeding larval stages. While the effectiveness of tebufenozide, in this capacity, is understood, management programs of other lepidopteran pests have demonstrated the effectiveness of tebufenozide application when utilized against other life stages. Here, we investigated the toxicity of topically-applied tebufenozide to *C. fumiferana* pupae to determine if such a strategy could be feasible. We observed significant dose-dependent decreases in the likelihood of adult emergence, increases in the likelihood of pupal death or adult deformity at eclosion, and significant decreases in mean adult longevity. Estimated LD 50 (lethal dose) values for adult male and female *C. fumiferana* treated as pupae ≤ 4 days after pupation were approximately 1–3 and 2–3.5% ACI (active commercial ingredient) respectively. Estimated L-SD (lethal-sublethal) 50 doses for adult male and female *C. fumiferana* treated as pupae ≤4 days after pupation were <1, and <2% ACI, respectively. Mating success was also significantly lower in mating pairs containing adults treated as pupae. Although, the amounts required to cause appreciable pupal mortality were much higher than those currently applied operationally in the *C. fumiferana* system, our study illustrates the potential of tebufenozide to utilized against additional developmental stages in other lepidopteran pests.

## 1. Introduction

*Choristoneura fumiferana* (Clemens) (Lepidoptera: Tortricidae) is a significant eruptive defoliator of balsam fir [*Abies balsamea* (L.) Mill.] and white spruce [*Picea glauca* (Moench) Voss] in Canada and the United States. The life cycle of this species is typically one-year in length [1,2]. Eggs are laid on foliage beginning in mid- to late-summer with first-instar larvae hatching from eggs ~10 days afterwards. First-instar larvae are primarily non-feeding, and form hibernacula within which they overwinter and moult. Second-instar larvae emerge in the spring and establish themselves on 1-year-old foliage. After moulting into a third-instar, they begin feeding on current-year foliage where available. Most of the feeding damage that is associated with the larval stage is caused by late-instar larvae (L_4_–L_6_). Larvae pupate within or in close association to feeding shelters constructed from shoots bound together by silk, from which they emerge as adults. While males are active flyers, adult females will fly only when most of their egg complement has been deposited. Large amalgamations of flying adults can be dispersed by weather and storm fronts, leading to the movement of individuals and potential increases in local populations [3]. Tree defoliation by feeding larvae is dependent on a number of abiotic and biotic factors. However, annual defoliation on shoots and flowers leads to reductions in radial growth, height growth, and eventually top kill [1]. Affected trees are also increasingly susceptible to secondary infestation by other insects, disease infection, and wind breakage [4,5]. In Atlantic Canada, outbreaks may last for up to 15 years, with previous outbreaks affecting up to approximately 58 million hectares of forested regions [3,6]. Defoliation of spruce/balsam fir forests in this region by *C. fumiferana* has recently been increasing [7,8], with significant increases in both timber and economic losses being predicted for provinces, such as New Brunswick [9]. Consequently, vigorous detection and management strategies for monitoring and regulating *C. fumiferana* populations, with the goal of reducing and/or preventing defoliation in vulnerable forest regions, have been initiated [8].

Several direct management strategies for reducing defoliation by *C. fumiferana* exist [1,8], including the aerial application of the insect growth regulator (IGR) tebufenozide [10]. Tebufenozide is a bisacylhydrazine ecdysteroid agonist that mimics natural ecdysone (20-hydroxyecdysone) within the target insect [11,12]. When consumed by the larvae, tebufenozide binds to the ecdysone receptor in the gut and initiates the moulting process. Unlike the initiation via natural ecdysone, this moulting process is not completed, and is ultimately fatal, to the affected individual [11,13,14,15]. An advantage of these agonists over traditional insecticides, such as organophosphates is their high specificity against lepidopteran targets, while exhibiting low toxicity to non-targets from other orders [12,16]. Tebufenozide is toxic to numerous lepidopteran pests including *Cydia pomonella* (L.) (Lepidotera: Tortricidae) [17], *Lobesia botrana* (Denis & Schiffermüller) [18], *Lambdina fiscellaria fiscellaria* (Guenée) (Lepidoptera: Geometridae) [19], and *Spodoptera littoralis* (Boisduval) (Lepidoptera: Noctuidae) [20], and has demonstrated its versatility as an effective insecticide in both, forest and agricultural environments.

The target *C. fumiferana* developmental stage for tebufenozide application is late-larval. Specifically, significant mortality is limited to individuals between 0 and 3 days of the fifth-instar, and 0 and 2 days of the sixth-instar [21], although fatal moulting occurs in older fifth-instar larvae upon moulting into sixth-instar larvae. The aerial application of tebufenozide against later larval instars of *C. fumiferana* has been consistently shown to significantly reduce both defoliation and mean number of larvae per branch in treated stands [22,23,24,25]. Currently, our understanding of the effective mortality of direct application of tebufenozide against other life stages is largely unknown. In other tortricids, including *C. pomonella* (L). [26,27,28], *Grapholita molesta* (Busck) [29,30], *Argyrotaenia velutinana* (Walker) [31] and *Choristoneura rosaceana* (Harris) [32,33], the application to non-target host stages can cause significant reductions in both fecundity and fertility, and significant reductions in the ability of individuals to locate potential mates. Such detrimental effects on *C. fumiferana* fitness may be important in lowering populations and reducing defoliation in treated areas. The aim of our research was to determine the effects of topical application of tebufenozide on *C. fumiferana* pupae of different age (1–5 days after pupation) in relation to their metamorphosis into normal or deformed adults, and to longevity and mating capacity of emerged adult. The results of our research provide important information regarding the possible effectiveness of tebufenozide outside of its current utilization for *C. fumiferana.*

## 2. Materials and Methods

### 2.1. Insects and Bioassays

Second-instar ‘diapause strain’ laboratory-reared larvae (L2) (2018: GLFC:IPQL:Cfum CF6-F23, F07, F08; 2019 GLFC:IPQL:Cfum F14, CF13 F23) [34] were obtained from the Insect Production Services (IPS) of the Canadian Forest Service in Sault Ste. Marie, ON, Canada. Laboratory bioassays were carried out from September 2018 to August 2019 at the Atlantic Forestry Center, New Brunswick Canada. L2 were placed in a small plastic cup containing standard McMorran artificial diet [35]. Larvae fed on diet until they reached pupation (approximately 2 weeks), at which time the pupae were removed from diet cups, separated by sex, and placed in a glass Petri dish. Rearing conditions were 16hr:8hr (day:night), constant 23 °C, and relative humidity (RH) of 40–70%. Pupae were separated by age (0–5 days old) post pupation and were treated with tebufenozide (RH-5992 flowable formulation Mimic™-2F, Rohm and Haas C., Spring House PA) to monitor its effects on development. The concentration of active tebufenozide in this formulation was 24.3 µg/µL. For treatment pupae, 1 µL topical applications containing 0.1, 0.25, 0.5, 1.0, 2.0, 5.0, 10.0, or 20% dilutions of active Mimic™-2F commercial ingredient (ACI) were applied (Table 1). Dilutions were made in deionized water. Control pupae were treated with 1 µL of deionized water. A cohort of pupae was also treated separately with 1 µL of Triton-X solution (7% Triton X-100 and 6% Glycerine in deionized water), the primary emulsifying ingredient in the commercial Mimic™-2F compound. Each treatment X age combination, consisted of ≥ 10 individuals. Treatments were applied using a glass syringe to the dorsal surface of the thorax. Following treatment, pupae were held individually in polystyrine disposable Petri dishes (100 mm × 15 mm) lined with filter paper. Insects were monitored daily until their death or emergence of the adult. The number of adults who emerged with a wing deformity was recorded. Adult longevity was also recorded. Surviving adults were placed in mating pairs in Petri dishes with a conspecific of an identical pupal age of exposure and dose combination to investigate the effects of tebufenozide exposure on adult fitness. For all mating pairs, the bursa copulatrix was dissected to determine the presence or absence of a spermatophore after female death. Mating pairs were kept together until one or both of the insects died (approx. 5–14 days). Adults were kept in environmental conditions identical to those described for the larvae.

### 2.2. Statistics

Both the metamorphosis of the pupa into an apparently normal adult, and the death of the pupa or its evolution into an adult with deformed wings, were distinctly treated for male and female pupae as binary responses and were modelled with respect to the treatment and age of the pupa, at the time of the treatment application, by logistic regression analysis. Where treatment was a significant effect in these analyses, a subsequent logistic regression analysis where treatments were analyzed as levels was completed to identify specific treatment doses where likelihood was significantly different from the control groups. Sex-specific LD (lethal dose) and L-SD (lethal-sublethal dose) 50 and 90 values for each age cohort were calculated using a 2-parameter dose-response model in the ‘drc’ statistical package [36] in the ‘R’ programming language [37]. The effect of treatment on mean adult longevity was analyzed using a two-way Analysis of Variance (ANOVA) considering treatment and pupa age at treatment application as fixed factors. As there were few moths eclosing to adult after treatments ˃ 2.0 ACI, only moths treated with 0%, 0.1%, 0.25%, 0.5%, and 1.0% ACI and Triton-X solution were included in these analyses. Finally, a generalized linear model (‘glm’, link = “binomial”) also using treatments and age at application as fixed factors was used to analyze the proportions of females containing a spermatophore after placement within mating pairs. Insufficient numbers for analysis were found in treatment groups ≥ 2% ACI. Therefore, this analysis only included treatment groups of ≤ 1% ACI as well as Triton-X and control groups. Specific sample sizes have been included in the figures and tables where applicable. These analyses were also carried out in the ‘R’ programming language [37]. An α = 0.05 was assumed as the level of significance for all statistical analyses. 

## 3. Results

**Figure 1:**
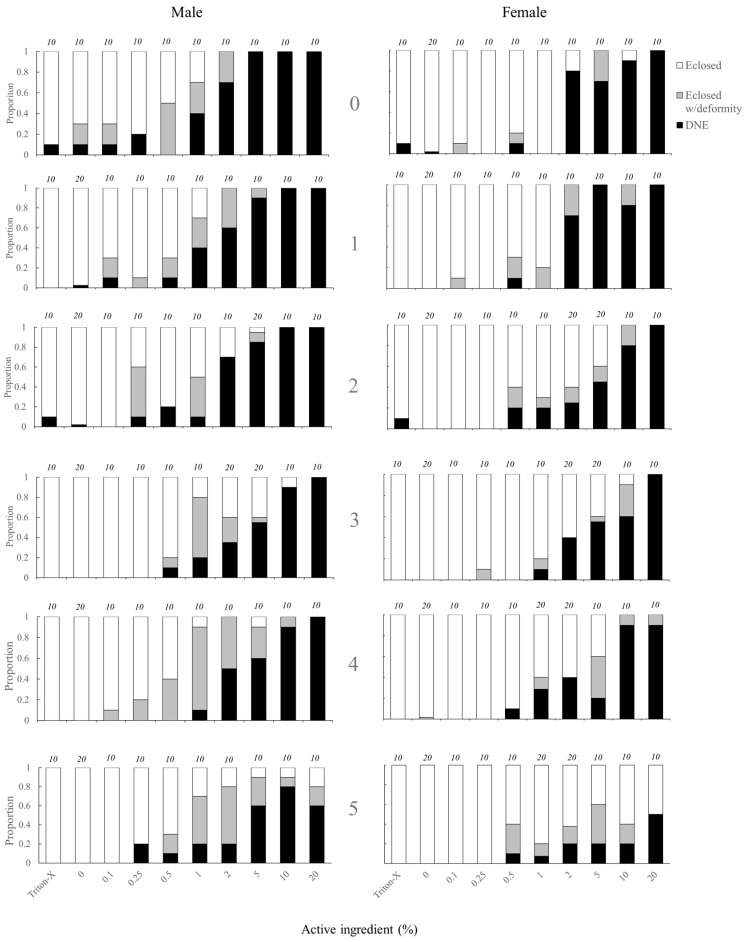
Age-specific proportions of *C. fumiferana* eclosing to an adult, eclosing to an adult with a deformity, or not eclosing (‘did not eclose’, DNE) following treatment with Triton-X, water, or various doses of tebufenozide (Mimic™). Age (indicated by 0, 1, 2, 3, 4, and 5 within figure) represents the number of days after pupation at which the individual was treated. Numeric values above data bars represent the number of individuals (‘n’) included in each treatment x age combination.

The likelihood of adult eclosion was significantly affected by the topical application of tebufenozide to pupae in both male and female individuals (Figure 1, Table 2). Treatment applications of ≥ 1.0% ACI and ≥ 2.0% ACI caused significant decreases in the likelihood of adult eclosion for males and females respectively (Figure 1, Table 3). There was also a significant effect of age on likelihood of adult eclosion, with younger individuals less likely to eclose than older individuals. An interaction between age and treatment for females was observed, with the likelihood to eclose to an adult being slightly higher in 4–5-day old pupae treated with higher % ACI treatments than in younger (0–3-day old) pupae (Figure 1, Table 3). The likelihood of an individual either failing to eclose to an adult or eclosing to an adult with a deformity was also significantly affected by the application of tebufenozide (Figure 1, Table 2). For males, significant reductions were observed in all groups ≥ 0.1% ACI except 0.25% ACI. Significant reductions for females occurred at 0.5% ACI and ≥ 2.0% ACI (Figure 1, Table 3). There was no significant effect of age on the likelihood of failing to eclose to an adult or eclosing to an adult with a deformity (Table 3).

Estimated LD (lethal dose) 50 values for adult male and female *C. fumiferana* treated as pupae ≤ 4 days after pupation were 1.093 ± 0.235 to 2.943 ± 0.601% AI and 4.225 ± 1.527 to 9.357 ± 3.087% AI for males and females respectively (Table 4). LD50 values for 5-day old pupal males and females were higher (5.020 ± 1.192% ACI and 26.35 ± 19.19% ACI respectively). Estimated LD90 values for males and females both increased with age, varying from 4.225 ± 1.527 to 9.357 ± 3.087% ACI and 7.230 ± 2.464 to 27.53 ± 15.65% ACI for males and females aged 0–4-day old respectively. Estimated LD90 values for 5-day old pupae of both sexes were ˃ 40% ACI. Estimated L-SD (lethal-sublethal) 50 doses for males varied from 1.895 ± 0.742 to 4.168 ± 1.641% ACI in 0–4-day old pupae (Table 5). L-SD90 for 5-day old pupae was higher (8.635 ± 4.700% ACI). L-SD90 values for 0–4-day old females were higher than those for males (2.727 ± 0.855 to 9.724 ± 3.81% ACI). L-SD90 for female 5-day old pupae was > 40% ACI.

Male longevity was significantly affected overall by treatment (F_4,335_ = 37.045, *p* < 0.001), age (F_1,335_ = 9.486, *p* = 0.002), and treatment x age (F_4,335_ = 2.615, *p* < 0.035) (Table 6). Female longevity was significantly affected by treatment (F_4,392_ = 15.534, *p <* 0.001) and age (F_1,392_ = 6.794, *p* = 0.01). Age-specific analyses showed the mean adult longevity in males was significantly lower in 1-, 2-, and 4-day old pupae treated with 0.25% ACI and 1.0% ACI. Significant reductions in mean longevity were also found in 0-day old males treated with 0.5% ACI and 1.0% ACI, and 2-day old males treated with 0.5% ACI. Treatment did not significantly reduce mean longevity in 3- and 5-day old male pupae. Mean female longevity was significantly reduced only in 3- and 5-day old pupae treated with 1.0% ACI. The treatment of pupae with Triton-X solution was never observed to significantly lower mean longevity when compared to untreated pupae.

**Figure 2:**
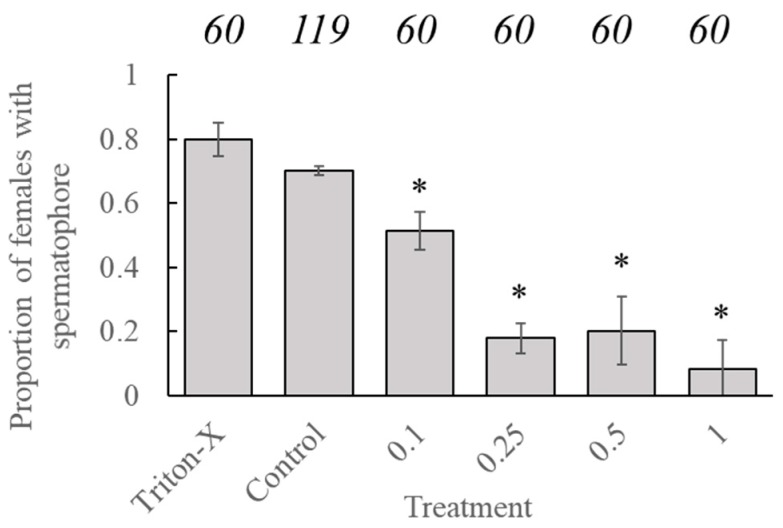
Proportion of adult *C. fumiferana* females containing a spermatophore after being paired with an adult male of an identical treatment dose. Numerical values above the bars represent the sample sizes (‘n’) for each treatment group. An ‘*’ represents a significant difference in the likelihood of a female containing a spermatophore as compared to the ‘control’ group (Generalized linear model, family = “binomial”, *p* < 0.05).

Statistical analysis showed no significant effect of age at application on the likelihood of females containing a spermatophore (*t* = −0.362, *p* = 0.7175). However, the likelihood of a female containing a spermatophore, after being placed in a mating pair with a conspecific male, was significantly reduced in all pairs where both insects had been treated with tebufenozide versus proportions associated with the control group (*t* = −6.723, *p* < 0.001) Factorial analysis indicated that significant reductions occurred in all groups containing treated individuals (Figure 2). There was no significant difference in the likelihood of females, containing a spermatophore for insects treated with Triton-X compared to untreated insects.

## 4. Discussion

The topical application of tebufenozide to pupae resulted in significant increases in the likelihood of individuals failing to eclose to an adult, or eclosing to an adult with a deformity. We also observed significant decreases in mean adult longevity, and reductions in the likelihood of male-female pairs successfully mating. The current utilization of tebufenozide is its application to *C. fumiferana* populations where the feeding larval stage is present. The effectiveness of tebufenozide against *C. fumiferana* larvae is well established [13,14,21,23,25]. However, the novel and important information presented here demonstrate the versatility of the larvicide tebufenozide as a topical insecticide against other lepidopteran life stages.

Treatment of male and female pupae resulted in significant reductions in the likelihood of adult eclosion, and the increased in the likelihood of adult eclosing with wing deformities or death. Sundaram et al. [36] analyzed the rates of pupal death in individuals intrahoemically injected with varying amounts of tebufenozide, and similarly found a dose-dependent relationship. The same authors also observed increases in the number of adults emerging with wing deformities, including increased disruption of wing scale deposition, and degeneration of wing epithelial cells [38]. Macro-level comparisons of deformed adults in this study showed a resemblance with those of Sundaram et al. [37], suggesting similar deformation modalities. Histological analyses are necessary to confirm if cellular deformities are consistent between these studies. The possession of wing deformities can have a negative effect on individual fitness, as deformed adult moths, specifically males, require the ability to engage in sustained flight to locate calling conspecific females. An inability to engage in flight and locate females can significantly affect a moth’s fitness. Dispersion is also an important ability for females to utilize after her egg compliment has been depleted if she is to locate suitable oviposition sites [3]. Wing deformation induced by exposure to insect growth regulators is not limited to *C. fumiferana,* as such effects have been observed in *Manduca sexta* (L.) (Lepidoptera: Sphingidae) [39], *Platynota idaeusalis* (Walker) (Lepidoptera: Totricidae) [40], and *Spodoptera exigua* (Hübner) (Lepidoptera: Noctuidae) [41]. Interestingly, both [10] and [32] observed that surviving adult *C. fumiferana* exposed to tebufenozide as larvae rarely had wing deformities. It is possible that dosages used in both our study and [37] were higher than those used in larval bioassays, resulting in higher numbers of deformed adults versus those observed in larval-focused studies.

While some studies have shown similar impacts on adult longevity, such as in *Ephestia kuehniella* Zeller (Lepidoptera: Pyralidae) [42] and *S. littoralis* [43], other studies analyzing longevity in surviving individuals have provided mixed results. In some cases, mean adult longevity may not be significantly affected by exposure [44], while in others, longevity may be significantly increased [45]. The mechanism by which adult longevity is lower in exposed individuals was not quantified in this study. However, the aforementioned disruptions to scale deposition and epithelial cell formation on the wings demonstrate that important effects on normal adult cuticle formation can be induced during pupal exposure. A closer inspection of adult scale deposition on the body, in treated individuals could identify potential factors related to cuticle formation, that may be related to reduced adult longevities.

Proportions of females, containing a spermatophore after being paired with a male of similar age and tebufenozide treatment, were significantly lower than females in mating pairs containing either untreated insects or those treated with Triton-X solution. This reduction in mating success agrees with predicted and observed mating success (also denoted by the number of females containing a spermatophore) in *C. fumiferana* adults treated as larvae as previously determined [11]. While, the mechanism by which mating success was disrupted was not determined in our study, or by Dhadialla et al. [11], other research suggests that applications to adult males cause a disruption in their ability to locate conspecific females. Hassan et al. [45] observed that adult male *C. pomonella* treated with another insect growth regulator, methoxyfenozide, were less responsive to calling adult females and synthetic pheromone sources. Consequently, these males mated significantly less than untreated adult males. Similar effects have been seen *Grapholita molesta* (Busck) [30], and *Argyrotaenia velutiana* (Walker) (Lepidoptera: Tortricidae) [33]. Another possible explanation may be a disruption to male spermatogenesis following exposure during the immature stages. In the noctuid *Spodoptera litura* Fabricius [46], treated males were found to have significant decreases in sperm production, including lower amounts of apryne and eupryne sperm being released into the reproductive tract. The transfer of sperm bundles to females during mating was also significantly reduced. Similar disruptions in sperm release after treatment with tebufenozide in adult males have also been observed in *Lymantria dispar* L. (Lepidoptera: Lymantriidae) [46]. Such effects on spermatogenesis and/or sperm transfer may be occurring in *C. fumiferana*, though further research is necessary to determine this. 

The ingestion of ≤ 0.03 and 0.06 µg per individual has been demonstrated to cause approximately 50%, and 90% mortality in sixth-instar, respectively [47]. Our results demonstrate that the topical application of tebufenozide requires amounts of active ingredient per individual four to eight times higher than those for larvae, in order to cause significant increases in pupal death. This precludes the possibility of tebufenozide application to pupae based on economic considerations alone. The direct consumption of the tebufenozide has been shown to be significantly more effective in ensuring a lethal dose is transmitted to the insect than topical application [45]; however, we observed that doses < 0.03 µg per insect were sufficient to disrupt mating in individuals exposed as pupae. Although, lower than the amounts necessary to theoretically prevent adult eclosion from a *C. fumiferana* pupae, the sublethal application of a compound, such as tebufenozide may possibly have similar effects on future L2 counts given that oviposition is similarly reduced, compared to directly eliminating the adult cohort. Interestingly, topical applications of tebufenozide dissolved in an aqueous solutions caused no larval mortality even up to 10 µg per insect [45], while we observed significant increases in pupal mortality and/or adults with deformities at 0.024–0.486 µg per insect. This suggests that *C. fumiferana* pupae may be more vulnerable to topical application than larvae. However, the application of a lethal or sublethal dose will be hindered by the likelihood of pupae residing within protected feeding shelters on the host tree. In combination with the economic costs associated with increased amounts required with an aerial application, it is clear that tebufenozide applications to pupae, with the goal of causing additional compensatory mortality, is not feasible. These results do, however, provide an additional example of IGRs being lethal to life stages beyond the target larvae. Our results, while not applicable within the *C. fumiferana* system, may be of importance in ascertaining the potential versatility of tebufenozide and IGRs in other population management programs involving lepidopteran pests.

## 5. Conclusions

Our results demonstrate that topical application of tebufenozide can cause reductions in adult emergence from treated pupae and increases in the incidence of emerging adults with deformities. Emerging adults topically treated as pupae also had reductions in mating success and some significant decreases in mean longevities when compared with untreated individuals. While the practical aspects of direct topical tebufenozide application to *C. fumiferana* are not economically feasible given the higher amounts required to effectively treat individuals and the largely protected pupal stage, this research serves as an important example of the potential applicability of insect growth regulators when applied to additional host stages of a target tortricid pest species. 

## Figures and Tables

**Table 1:** Quantities of active tebufenozide ingredient diluted from RH-5992 flowable formulation Mimic™-2F [24.3 µg/µL], Rohm and Haas C., Spring House PA in used in 1 µL topical applications on *C. fumiferana* pupae.

Percent (%) Dilution	0.1	0.25	0.5	1.0	2.0	5.0	10.0	20.0
Active tebufenozide quantity (µg)	0.024	0.061	0.122	0.243	0.486	1.215	2.430	4.860

**Table 2:** Main effects of tebufenozide dosage (% ACI) and pupal age at treatment on likelihood of the adult eclosion of a normal adult and likelihood of pupal death/adult eclosion with a deformity in *C. fumiferana* pupae. A ‘*’ represents a significant effect on likelihood (*p* = 0.05).

		Adult Eclosion		Adult Failed to Eclose/Deformed	
Sex	Effect	t-Value	*p*	t-Value	*p*
Male	Age	2.708	0.007 *	1.611	0.108
	Treatment	−15.360	<0.001 *	−10.899	<0.001 *
	Age x Treatment	2.223	0.026 *	1.024	0.306
Female	Age	2.499	0.0126 *	1.870	0.0617
	Treatment	−16.565	<0.001 *	−12.731	<0.001 *
	Age x Treatment	2.757	0.006 *	0.248	0.8043

**Table 3:** Effects of treatment dosage on a) likelihood of adult eclosion; and b) likelihood of pupal death/adult eclosion with a deformity (Multiple logistic regression; ‘*’ represents a significant difference in likelihood versus control (untreated) pupae (*p =* 0.05)).

		Adult Eclosion		Adult Failed to Eclose/Deformed	
Sex	Treatment	t-Value	*p*	t-Value	*p*
Male	0.1	−0.490	0.6246	−2.438	0.0150 *
	0.25	0.447	0.6550	0.445	0.6563
	0.5	0.490	0.6246	−4.389	<0.001 *
	1.0	−3.427	<0.001 *	−6.339	<0.001 *
	2.0	−6.365	<0.001 *	−9.265	<0.001 *
	5.0	−9.303	<0.001 *	−9.265	<0.001 *
	10.0	−8.323	<0.001 *	−8.290	<0.001 *
	20.0	−9.303	<0.001 *	−9.265	<0.001 *
Female	0.1	0.223	0.8238	0.185	0.8530
	0.25	0.213	0.8313	0.177	0.8532
	0.5	−0.846	0.3976	−2.484	0.0132 *
	1.0	0.223	0.8238	0.185	0.8530
	2.0	−8.329	<0.001 *	−8.711	<0.001 *
	5.0	−9.482	<0.001 *	−11.378	<0.001 *
	10.0	9.398	<0.001 *	−7.822	<0.001 *
	20.0	−10.467	<0.001 *	−8.711	<0.001 *

**Table 4:** Estimated lethal dose (LD) (% ACI) to achieve 50 and 90% of mortality in *C. fumiferana* pupae on which tebufenozide had been topically applied.

	Age	LD50	Lower	Upper	LD90	Lower	Upper
Male	0	1.093 ± 0.235	0.633	1.554	4.225 ± 1.527	1.232	7.218
	1	1.369 ± 0.292	0.798	1.941	5.122 ± 1.831	1.534	8.710
	2	1.566 ± 0.312	0.955	2.176	5.700 ± 1.741	2.287	9.113
	3	2.233 ± 0.738	0.786	3.680	7.773 ± 3.975	−0.019	15.56
	4	2.943 ± 0.601	1.765	4.122	9.357 ± 3.087	3.307	15.41
	5	5.020 ± 1.912	1.272	8.768	>40.00	NA	NA
Female	0	2.153 ± 0.446	1.278	3.027	7.230 ± 2.464	2.401	12.06
	1	1.980 ± 0.389	1.217	2.742	5.913 ± 1.899	2.191	9.635
	2	2.452 ± 0.838	0.810	4.094	12.03 ± 6.551	−0.808	24.87
	3	5.640 ± 1.778	2.156	9.124	19.58 ± 7.845	4.210	34.96
	4	3.393 ± 0.820	1.786	5.000	27.53 ± 15.65	−3.137	58.20
	5	26.35 ± 19.19	−11.26	63.96	>40	NA	NA

**Table 5:** Estimated lethal-sublethal dose (L-SD) (%ACI) to achieve 50 and 90% pupal death/eclosed to an adult with a deformity in *C. fumiferana* pupae on which tebufenozide had been topically applied.

	Age	LD50	Lower	Upper	LD90	Lower	Upper
Male	0	0.397 ± 0.098	0.204	0.589	1.895 ± 0.742	0.440	3.350
	1	0.511 ± 0.117	0.281	0.740	2.183 ± 0.815	0.585	3.781
	2	0.687 ± 0.174	0.345	1.027	4.168 ± 1.641	0.952	7.384
	3	0.888 ± 0.223	0.451	1.325	2.884 ± 1.494	−0.045	5.813
	4	0.481 ± 0.102	0.280	0.681	1.729 ± 0.594	0.565	2.893
	5	0.906 ± 0.268	0.381	1.431	8.635 ± 4.700	−0.578	17.85
Female	0	1.471 ± 0.312	0.860	2.081	5.373 ± 1.905	1.640	9.106
	1	0.933 ± 0.177	0.585	1.281	2.727 ± 0.855	1.051	4.404
	2	1.081 ± 0.291	0.508	1.653	3.416 ± 1.84	−0.195	7.027
	3	2.295 ± 0.762	0.802	3.788	8.701 ± 4.47	−0.068	17.48
	4	1.970 ± 0.347	1.291	2.650	9.724 ± 3.81	2.264	17.18
	5	8.206 ± 4.485	−0.583	17.00	>40	NA	NA

**Table 6:** Mean longevity of adult male and female *C. fumiferana* treated with tebufenozide solutions during pupal development. Age represents the number of days after pupation at which 1µL of a tebufenozide solution was topically applied to the individual. An ‘*’ represents a mean longevity that is significantly lower than the mean longevity of the associated control group (Tukey HSD pair-wise comparison, *p* < 0.05).

		Triton-X		Control		0.1%ACI		0.25%ACI		0.5%ACI		1.0%ACI	
	Age	n	$\bar{x}$ ± SE	n	$\bar{x}$ ± SE	n	$\bar{x}$ ± SE	n	$\bar{x}$ ± SE	n	$\bar{x}$ ± SE	n	$\bar{x}$ ± SE
♂	0	10	6.25 ± 0.25	28	6.89 ± 0.318	9	5.66 ± 0.577	7	5.57 ± 0.649	9	2.44 ± 0.709 *	6	3.83 ± 0.792 *
	1	10	6.4 ± 0.306	28	7.07 ± 0.388	9	7.0 ± 0.441	9	4.33 ± 0.577 *	9	5.44 ± 0.556	6	3.5 ± 1.15 *
	2	10	6.2 ± 0.291	20	7.3 ± 0.411	10	6.6 ± 0.221	9	4.22 ± 0.434 *	8	4.12 ± 0.742 *	9	4.11 ± 0.841 *
	3	10	6.5 ± 0.477	20	7.25 ± 0.369	10	5.4 ± 0.909	7	6.28 ± 0.359	9	5.11 ± 0.633	8	2.75 ± 0.366
	4	10	6.8 ± 0.553	20	8.05 ± 0.569	10	6.5 ± 0.401	10	5.0 ± 0.699 *	10	5.7 ± 0.578	9	2.89 ± 0.754 *
	5	10	7.1 ± 0.348	19	7.36 ± 0.514	10	6.2 ± 0.573	10	5.3 ± 0.597	9	6.55 ± 0.689	9	5.25 ± 0.648
♀	0	10	11.3 ± 0.726	27	9.33 ± 0.591	10	8.1 ± 0.746	9	10.8 ± 1.04	9	9.0 ± 1.27	10	8.5 ± 1.27
	1	10	9.5 ± 0.637	30	9.93 ± 0.738	10	10.7 ± 0.831	8	12.0 ± 1.31	9	9.11 ± 0.858	10	8.2 ± 0.706
	2	10	10.1 ± 0.564	30	10.8 ± 0.498	10	11.2 ± 0.871	10	12.6 ± 0.895	8	10.0 ± 0.803	8	6.75 ± 0.749
	3	10	10.9 ± 0.956	30	9.9 ± 0.297	10	9.9 ± 0.786	10	13.0 ± 0.778	10	10.2 ± 1.13	9	7.33 ± 0.707 *
	4	10	12.1 ± 0.567	30	10.6 ± 0.80	10	10.4 ± 0.862	10	11.0 ± 0.817	9	10.0 ± 1.03	9	9.3 ± 0.604
	5	10	10.9 ± 0.809	29	11.0 ± 0.658	10	10.7 ± 0.813	9	12.8 ± 0.852	9	11.1 ± 0.971	9	6.89 ± 1.31 *
